# Effective use of oral tofacitinib and phototherapy in a patient with concomitant alopecia areata, vitiligo, and plaque and inverse psoriasis

**DOI:** 10.1002/ccr3.2759

**Published:** 2020-02-27

**Authors:** Mahroo Tajalli, Soodeh Kabir, Terrence M. Vance, Abrar A. Qureshi

**Affiliations:** ^1^ Department of Dermatology Warren Alpert Medical School Brown University Providence RI USA; ^2^ Comprehensive Cancer Center University of Maryland Baltimore MD USA; ^3^ Department of Epidemiology School of Public Health Brown University Providence RI USA

**Keywords:** alopecia areata, inverse psoriasis, phototherapy, plaque psoriasis, tofacitinib, vitiligo

## Abstract

This case presentation suggests that tofacitinib combined with phototherapy may be an effective treatment option for patients with concomitant alopecia areata, vitiligo, and different phenotypes of psoriasis including plaque and inverse psoriasis.

## Graphical abstract

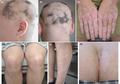

## 1 INTRODUCTION

Tofacitinib is a member of first‐generation Janus kinase (JAK) inhibitors which was initially studied as an antirejection agent in organ transplantation.^1^, ^2^ Recent studies have demonstrated the efficacy of tofacitinib in the treatment of multiple phenotypes of autoimmune skin conditions, including atopic dermatitis (AD), alopecia areata (AA), psoriasis, and vitiligo. The pathogenesis of all these diseases involves an immune dysregulation which can be targeted and reversed by the use of tofacitinib.^2^, ^3^ Here, we report a patient with concomitant AA, plaque and inverse psoriasis, and vitiligo who responded to treatment with tofacitinib.

## 2 CASE REPORT

**Figure 1:**
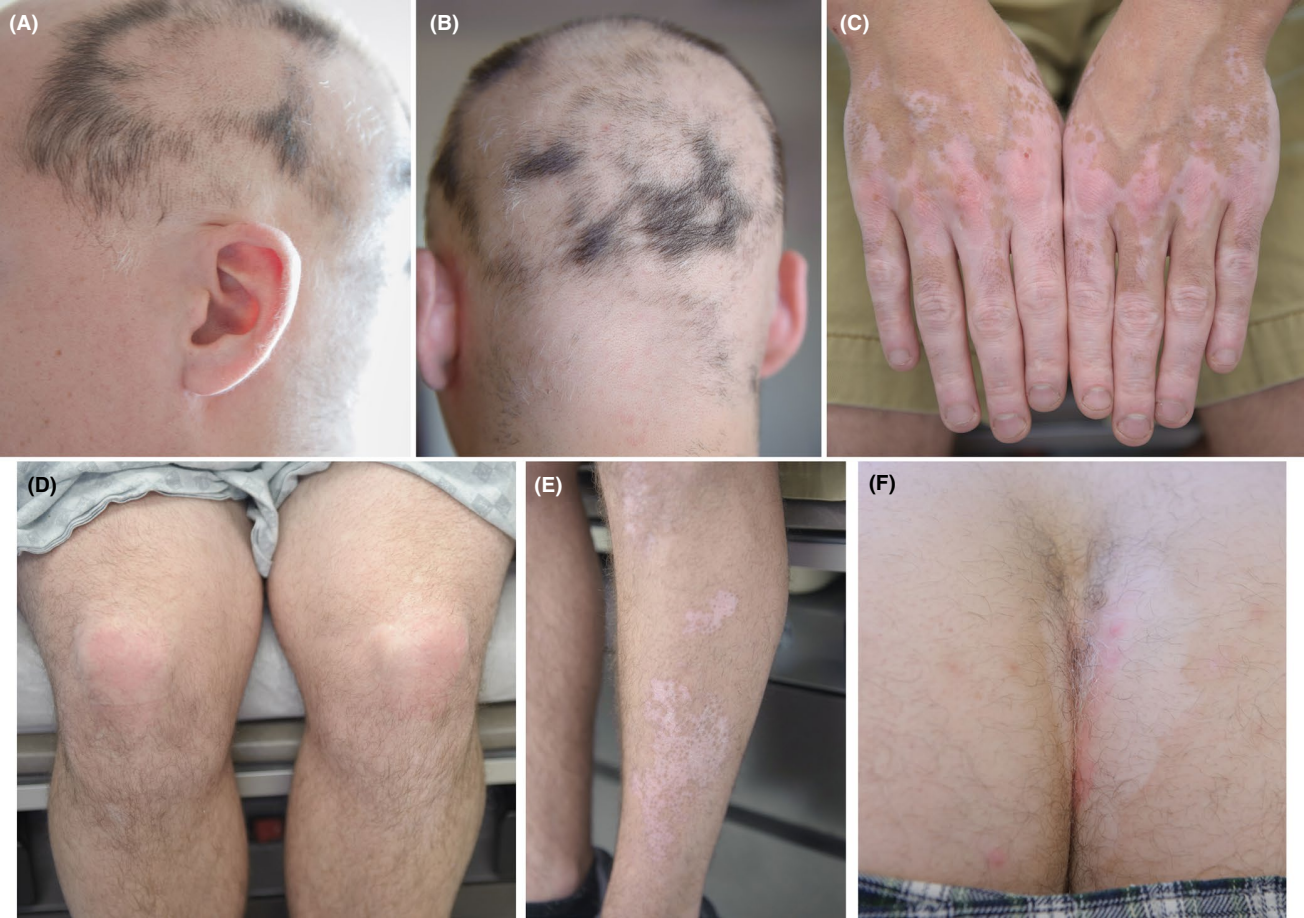
Clinical presentation of skin lesions before initiation of treatment with tofacitinib and phototherapy. A and B, Alopecia areata of the scalp. C, Vitiligo of dorsum of hands. D, Psoriasis of the knees. E, Vitiligo of the lateral aspect of the leg. F, Psoriasis and vitiligo of the gluteal cleft

A 30‐year‐old gentleman presented to our clinic complaining of hair loss on the scalp for <1 month. The patient also had depigmented patches on the face, chest, both elbows, dorsum of hands, and both legs for 4 years and complained of erythematous, scaly lesions on both elbows and knees and erythema and pruritus of both axillae and the intergluteal cleft for 8 years. Family history was significant for vitiligo in his grandfather and thyroid disease in his father and grandfather. Examination revealed multiple patches of nonscarring alopecia on the scalp; and based on history and physical examination, he was diagnosed with AA, nonsegmental generalized vitiligo, and plaque and inverse psoriasis. Thyroid studies were normal. He previously had received two sessions of phototherapy for vitiligo with no improvement. No treatment was received for AA and psoriasis. We prescribed intralesional triamcinolone (ILT) injection for AA, topical steroid and tacrolimus for vitiligo, and topical steroid for psoriasis. One month later, he showed some hair regrowth in previously injected areas of AA, however, new areas of hair loss had developed; improvement of psoriasis was modest; and vitiligo lesions were unchanged. After two rounds of ILT for AA, little to no response was demonstrated and over the next few months AA progressed to alopecia universalis, involving large areas of the scalp, eyebrows, eyelashes, and body hair (Figure 1). At this point, we prescribed oral tofacitinib and phototherapy to treat all three skin disorders. He was started on oral tofacitinib 5 mg twice daily along with narrowband ultraviolet‐B (NB‐UVB) phototherapy three times a week. Following treatment, all psoriatic lesions improved after 1 week of treatment and regrowth of nearly all scalp and body hair occurred within 3 months. All vitiligo lesions improved with perifollicular repigmentation after three months of initiation of treatment. Despite our advice to receive the flu vaccine at the initiation of the treatment, the patient declined and following 4 months of treatment, due to 3‐4 episodes of headache and flu‐like symptoms, he self‐discontinued tofacitinib for 1 month. Therefore, at his next visit, 5 months after initiation of treatment, we restarted tofacitinib with a lower dose of 5 mg daily. He received the flu vaccine at this time.

**Figure 2:**
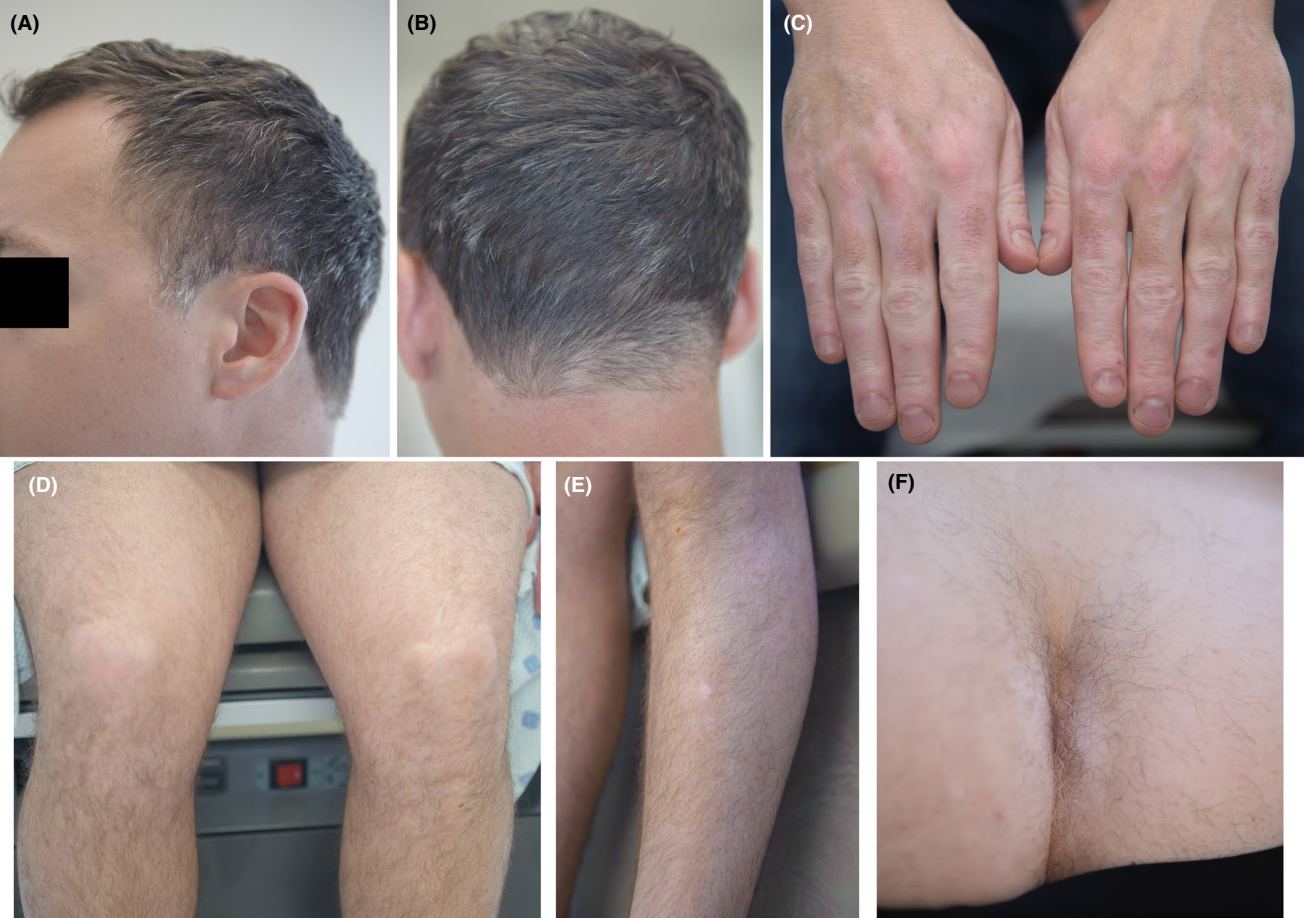
Maintenance of improvement of skin disorders one year after initiation of the treatment with tofacitinib and phototherapy. A and B, Nearly complete regrowth of hair on the scalp. C, E, and F, Improvement of vitiligo lesions. D and F, Complete resolution of psoriasis lesions

At this lower dose of tofacitinib, psoriasis and AA remained in remission and the vitiligo lesions continued to improve over the course of more than 1‐year of follow‐up (Figure 2).

## 3 DISCUSSION

Tofacitinib is a JAK 1/3 inhibitor that has been approved for the treatment of rheumatoid arthritis, psoriatic arthritis, and ulcerative colitis.^3^ However, it has also been used for immune‐mediated inflammatory skin disorders such as psoriasis, vitiligo, AA, and AD with varying degrees of efficacy and safety.^4^ For instance, in a retrospective study of tofacitinib use in 13 patients with AA, a scalp hair regrowth rate >50% was reported in 53.8% of patients.^5^ On the other hand, in a phase II study of tofacitinib in 66 patients with AA, Crispin et al reported a 32% clinical response (>50% improvement in Alopecia severity score).^6^ Comparable efficacy profiles have been reported for tofacitinib in treating psoriasis and vitiligo.^4^

With respect to its safety, tofacitinib use in AA patients has been associated with grade I or II infections. Except for a tendency toward causing more herpes zoster infection in psoriasis patients, the rate of other adverse events associated with the use of tofacitinib for the treatment of vitiligo or psoriasis is comparable to other systemic therapies.^3^, ^6^, ^7^

Although tofacitinib is the most studied JAK inhibitor in moderate to severe plaque psoriasis,^4^ evidence of its efficacy in the treatment of inverse psoriasis is lacking. In this case presentation, both plaque and inverse psoriasis of the patient responded to tofacitinib within 1 week and achieved a long‐term remission. This suggests that tofacitinib may be useful in the treatment of psoriasis resistant to topical agents. On the other hand, perifollicular repigmentation was observed in the patient's vitiligo patches in response to tofacitinib. A possible explanation could be that concomitant tofacitinib and NB‐UVB act synergistically, which is supported by studies that have shown that JAK inhibitors are more effective when combined with phototherapy.^8^, ^9^

The presented patient had a combination of AA, psoriasis, and vitiligo, and tofacitinib was effective in the treatment of all the 3 disorders. To our knowledge, tofacitinib use has not yet been reported in a patient having AA, psoriasis, and vitiligo. However, it has been used in patients with other combinations of autoimmune skin disorders. For instance, Vu et al reported a patient with AD, vitiligo, and AA who was treated with tofacitinib 5 mg twice daily. After 6 months of treatment, the patient experienced a marginal, partial, and complete response of vitiligo, AA, and AD, respectively.^10^ Other immunomodulators, however, have been tried in patients with a similar combination of disorders. One example is a patient reported by Elkady et al, who had concomitant AA, psoriasis, and vitiligo. The use of ustekinumab in this patient was associated with a complete remission in psoriasis in 16 weeks and partial remission in AA and vitiligo.^11^

Given that the simultaneous presentation of autoimmune skin disorders is not uncommon in clinical practice, it is of paramount importance to find the best treatment in such specific situations. This is particularly important from the decision‐making standpoint, when it comes to discussing the cons and pros of different treatment options for patients having multiple disorders. This case presentation can be a nidus for further studies by introducing tofacitinib as an effective and safe treatment option in this context. Until more robust evidence is available to guide the use of tofacitinib in patients with multiple cutaneous autoimmune disorders, preliminary data suggest that its off‐label use may be a possible therapeutic option.

## CONFLICT OF INTEREST

None declared.

## AUTHOR CONTRIBUTIONS

MT: contributed to accessing to the data and writing of the manuscript, and served as corresponding author. SK: contributed to writing of the manuscript. TMV: contributed to writing and revision of the manuscript. AAQ: contributed to revision and editing of the manuscript.
